# Decay-Accelerating Factor Restrains Complement Activation and Delays Progression of Murine cBSA-Induced Membranous Nephropathy

**DOI:** 10.34067/KID.0000000000000122

**Published:** 2023-04-08

**Authors:** Kelly L Budge, Alberto Verlato, Sofia Bin, Fadi E. Salem, Laura Perin, Gaetano La Manna, Gianluigi Zaza, Enrico Fiaccadori, Chiara Cantarelli, Paolo Cravedi

**Affiliations:** 1 Department of Medicine, Icahn School of Medicine at Mount Sinai, New York, New York; 2 Renal Unit, Department of Medicine, University Hospital of Verona, Verona, Italy; 3 IRCCS Azienda Ospedaliero-Universitaria di Bologna, UO di Nefrologia Dialisi e Trapianto, Bologna, Italy; 4 CIRI Scienze della Vita e Tecnologie per la Salute - Alma Mater Studiorum Università di Bologna, Bologna, Italy; 5 Department of Pathology and Molecular and Cell Based Medicine, Icahn School of Medicine at Mount Sinai, New York, New York; 6 Division of Urology, GOFARR Laboratory, Saban Research Institute, Children's Hospital Los Angeles, University of Southern California, Los Angeles, California; 7 Nephrology, Dialysis and Transplantation Unit, University of Foggia, Foggia, Italy; 8 Dipartimento di Medicina e Chirurgia Università di Parma, UO Nefrologia, Azienda Ospedaliero-Universitaria di Parma, Parma, Italy

**Keywords:** C3a, C3aR, DAF, classical pathway, complement, membranous nephropathy

## Abstract

**Key Points:**

- In a murine model of cationic bovine serum albumin (cBSA)–induced membranous nephropathy (MN), complement regulator decay-accelerating factor is upregulated and restrains complement activation.
- Studies using genetic deletion or pharmacological antagonism of C3aR indicate that the main effector mechanism of complement activation in cBSA-induced MN is C3a/C3aR signaling.
- C3a formation and/or C3aR-mediated signaling represent promising targets for hypothesis-driven therapies for MN.

**Background:**

Complement activation is believed to play a major pathogenic role in membranous nephropathy (MN), but its effector mechanisms are still unclear. Even less investigated is the role of podocyte-expressed complement regulators, including decay-accelerating factor (DAF) in disease pathophysiology.

**Methods:**

We induced MN by serial injections of cationic bovine serum albumin (cBSA) in WT, DAF^−/−^, and C3aR^−/−^ BALB/c mice and measured disease severity (by albuminuria, BUN, serum albumin, and glomerular histologic changes) and signs of complement activation in the glomeruli (immunofluorescence for C1q, C3b, and membrane attack complex). We also treated DAF^−/−^ mice with cBSA-induced MN with a selective C3aR antagonist and measured the same readouts.

**Results:**

cBSA-induced MN was associated with increased glomerular expression of DAF. Genetic deletion of DAF resulted in increased complement activation and higher disease severity than in WT animals. Treating cBSA-injected DAF^−/−^ mice with a C3aR antagonist reduced disease severity. Similarly, C3aR^−/−^ animals were protected from cBSA-induced MN, despite IgG deposition in the glomeruli and complement activation. Evidence of C1q and C3b deposition in the glomeruli of these mice suggest that IgG-cBSA immune complex formation in the glomeruli activates complement through the classical pathway.

**Conclusions:**

On cBSA-induced injury, podocytes upregulate DAF expression, which restrains complement activation. However, after prolonged injury, complement activation overcomes DAF regulatory effects leading to the formation of soluble anaphylatoxin C3a that, by signaling through C3aR, promotes glomerular injury and cBSA-induced MN disease progression. Considering the growing number of complement targeting therapies, our findings may have major translational effect on the treatment of patients with MN.

## Graphical abstract

**Figure:**
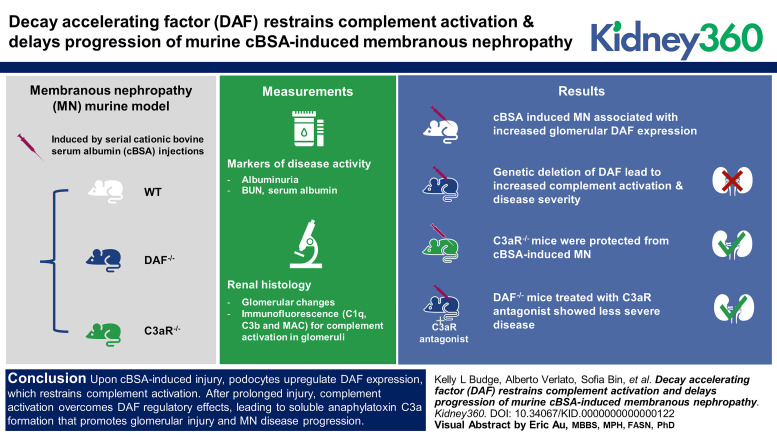

## Introduction

Membranous nephropathy (MN) is a major cause of nephrotic syndrome.^1^ Most of MN cases in adults are due to autoantibodies targeting podocyte-expressed antigens, including primarily the M-type phospholipase A2 receptor (PLA_2_R).^2^ More recently, additional target podocyte antigens have been identified, including, among others, thrombospondin type-1 domain-containing 7A (THSD7A),^3^ exostosin 1/2 (EXT1/EXT2),^4^ semaphorin 3B (SEMA3B),^5^ nerve epidermal growth factor-like 1 (NELL1),^6^ neural cell adhesion molecule 1 (NCAM1),^7^ protocadherin 7 (PCDH7),^8^ high temperature requirement A serine peptidase 1 (HTRA1),^9^ and netrin G1 (NTNG1).^10^

Nonpodocyte circulating antigens can also be involved in MN pathogenesis.^11^ In children with MN, both circulating cationic bovine serum albumin (cBSA) and anti-BSA antibodies have been found.^11^ In these cases, cBSA antigen colocalizes with immunoglobulin (Ig) G in subepithelial immune deposits, supporting the notion that cBSA serves as a planted antigen, and concomitant immunization against this antigen is responsible for the induction of MN.^12^

In both adult and childhood cases, the complement system is believed to play a key pathogenic role, but the exact mechanism has not been fully detailed. In particular, recent data indicate that anti-PLA_2_R IgG4, the main IgG subclass in MN, activates complement through the lectin pathway, but the effector mechanism is still unclear.^12^ While initial reports pointed at membrane attack complex (MAC) formation as the main effector mechanism,^13,14^ later experimental studies and clinical data with anti-C5 antibody seem to indicate that other complement-mediated effector mechanisms are at play, including C3a/C3a receptor (C3aR) signaling.^12,15^ However, data generated so far are limited to *in vitro* experiments with immortalized podocyte cell lines.^12^

Even less clear is the role of complement regulators in disease pathogenesis. Decay-accelerating factor (DAF/CD55) is a glycophosphatidylinositol-anchored protein that regulates complement activation on the surfaces on which it is expressed by accelerating the decay and by inhibiting the reformation of surface-bound C3 convertases, together restraining amplification of the complement cascade.^16^ DAF is expressed on podocytes^17^ and locally restrains complement-dependent podocyte injury that results in glomerulosclerosis.^18^ Our previous work in a model of focal segmental glomerulosclerosis also indicates that, on DAF cleavage, the main complement mechanism mediating podocyte injury is C3a/C3aR signaling,^18^ consistent with *in vitro* data by others.^12^

The role of DAF in MN has not been evaluated yet. In this study, we used the cBSA murine model of MN to test the hypothesis that DAF acts as a critical modulator of complement activation during disease and that C3a/C3aR signaling is the main effector mechanism responsible for complement-induced podocyte injury *in vivo*.

## Methods

### Mice and Procedures

BALB/c wild type (WT) and BALB/c C3aR^−/−^ mice were purchased from the Jackson Laboratory (Bar Harbor, ME). Previously generated B6 DAF^−/−^ animals (Dr. Heeger, Icahn School of Medicine at Mount Sinai, NY)^19^ were backcrossed 14 generations to BALB/c to produce BALB/c DAF^−/−^ animals.

The protocol for disease induction was adapted from Wu *et al.*^20^ Male mice of at least 20 g and aged 8 weeks were injected subcutaneously at the base of the tail with an emulsion of 0.2 mg cBSA (Chondrex, Woodinville, WA) with an equal volume of CFA (Sigma-Aldrich, St. Louis, MO). Two weeks after immunization, nephritis was induced with repeated retro-orbital injections of 0.1 mg cBSA every other day, over a 2-week period. Free water access and normal diet were maintained over the course of the study. Control mice received CFA and PBS (vehicle) instead of cBSA. Mice were sacrificed at 8 weeks postinduction (12 weeks after the first injection).

In selected experiments, C3aR antagonist (SB290157; Cayman Chemical, Ann Arbor, MI) or vehicle control was administered i.p. beginning the day of immunization at the dose of 10 mg/kg biweekly (powder dissolved in PBS and 10% DMSO) until euthanasia.

Animal study protocols were approved by the institutional animal care and use committee at Icahn School of Medicine at Mount Sinai (New York, NY; IACUC ID PROTO202000090). The reference number of the Office of Laboratory Animal Welfare approved Animal Welfare Assurance of Icahn School of Medicine at Mount Sinai is D16-00069 (A3111-01).

### Serum Anti-cBSA Antibodies

Serum samples were collected at sacrifice, and anti-cBSA IgG concentrations were measured using an ELISA, according to the previously described protocol.^21^ In brief, 96-well ELISA plates were coated with cBSA (0.5 *µ*g/well) at 4°C overnight. Anti-cBSA antibodies were detected using goat anti-mouse IgG (H+L) HRP-conjugated secondary antibody (1:10,000; Invitrogen, Carlsbad, CA, 31430) incubated for 1 hour at room temperature.

### Urinary Albumin and Creatinine

Albuminuria was monitored by weekly urine collection from individual mice before treatment and at weekly intervals until sacrifice. Urinary creatinine was quantified using commercial kits from Cayman Chemical. Urinary albumin was determined using a commercial assay from Bethyl Laboratories (Montgomery, TX). Urinary albumin excretion was expressed as the ratio of urinary albumin to creatinine.

### Serum Albumin and BUN

Serum was collected from all mice at the time of sacrifice. Serum albumin and BUN were measured using the colorimetric detection kit from Cayman Chemical and Thermo Fisher Scientific, respectively, according to the manufacturers' instructions.

### Renal Histology

Mice were anesthetized with a 100-*µ*l i.p. injection of a solution made of sterile ketamine (16 mg/ml) and xylazine (7 mg/ml) in PBS (Gibco, Thermo Fisher Scientific, Waltham, MA), and then, they received intracardiac perfusion of periodate-lysine-paraformaldehyde (Thermo Fisher Scientific) fixate at 4% in PBS at a rate of 8–10 ml/min. Kidneys were harvested and embedded in paraffin or frozen in optimal cutting temperature compound (Tissue-Tek O.C.T.; Sakura Finetek, Torrance, CA).

#### Light Microscopy

Paraffin-embedded kidney sections (3 *µ*m) were stained with periodic acid–Schiff (PAS) or Jones' stain (methenamine silver-PAS stain; Polysciences, Warrington, PA).

#### Immunofluorescence

O.C.T.-preserved cryosections (5 *µ*m thick) were washed with PBS for 15 minutes and then left for 60 minutes at room temperature with blocking solution (2% BSA, 2% FBS, and 0.2% fish gelatin in PBS). AffiniPure fab fragment goat anti-mouse IgG (Jackson ImmunoResearch, West Grove, PA) was subsequently applied for 3 hours, followed by incubation at 4°C overnight or at room temperature for 1 hour with specific primary antibodies against the following antigens: total anti-mouse IgG (1:32; Sigma, St. Louis, MO, F0257), C1q (1:25; Thermo Fisher Scientific, MA1-40045), C3b (1:50; Hycult Biotech, Plymouth Meeting, PA, HM1065), MAC (1:100; Abcam, Cambridge, MA, ab55811), DAF (1:50; BioLegend, San Diego, CA, 131802), and synaptopodin (1:5; Fitzgerald, Acton, MA, 10R-S125a). Sections were then washed and incubated with the appropriate secondary antibody for 60 minutes at room temperature: anti-mouse IgG antibody conjugated with Alexa Fluor 594 (1:200; Thermo Fisher Scientific), anti-rat IgG antibody conjugated with Alexa Fluor 488 (1:200; Thermo Fisher Scientific), and anti-rabbit IgG antibody conjugated with Alexa Fluor 488 (1:200; Thermo Fisher Scientific). Nuclei were counterstained with 4’,6-diamidino-2-phenylindole (DAPI) mounting media (ProLong Gold antifade reagent with DAPI, Invitrogen).

Images were acquired on a Zeiss widefield Axio Imager.Z2(M) and confocal LSM 880 Airyscan microscopes. Mean fluorescence intensity of various target antigens was quantified by using ImageJ software (National Institutes of Health, Bethesda, MD) within contour masks created on the synaptopodin-stained image to identify glomeruli.

### Statistics

We used one-way ANOVA on ranks (Kruskal–Wallis test) for multiple independent group comparisons and the Mann–Whitney test for two-group comparisons. We used two-way ANOVA (mixed-effects analysis) to compare urinary albumin/creatinine ratio values across multiple groups over time. A two-tailed *P* value <0.05 was regarded as statistically significant. All statistical analyses were performed using Prism, version 7, for Windows (GraphPad Software Inc.).

## Results

### The cBSA Murine Model of MN is Characterized by Complement Activation

**Figure 1:**
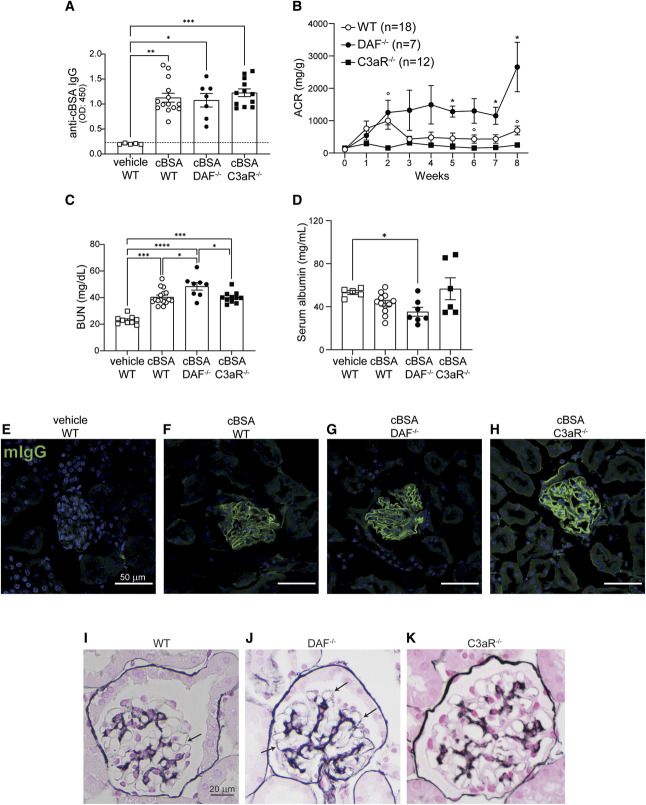
**Disease severity is altered by genetic deletions in the cBSA-induced MN model in BALB/c mice.** Serum concentration of anti-cBSA IgG antibodies in WT (*n*=14), DAF^−/−^ (*n*=7), and C3aR^−/−^ (*n*=12) BALB/c mice at 8 weeks after vehicle or cBSA injections (12 weeks after the initial cBSA immunization) (A). Urinary ACR at weekly intervals after cBSA injections in WT (*n*=18), DAF^−/−^ (*n*=7), and C3aR^−/−^ (*n*=12) (B); BUN (WT *n*=16, DAF^−/−^ *n*=8, C3aR^−/−^ *n*=11) (C) and serum albumin (WT *n*=18, DAF^−/−^ *n*=7, C3aR^−/−^ *n*=12) (D) at 8 weeks post-cBSA injections. Each data point represents a single mouse. Serum analyses were performed in a subset of mice with sufficient serum available. Representative images of total IgG staining in vehicle (E) and in cBSA-treated WT (F), DAF^−/−^ (G), and C3aR^−/−^ (H) BALB/c mice. Scale bars 50 *µ*m. Representative histological spike formation with methenamine silver staining for WT (I), DAF^−/−^ (arrows indicate glomerular basement membrane spikes) (J), and C3aR^−/−^ (K) mice at 12 weeks after the initial cBSA immunization. Scale bars 20 *µ*m. *P < 0.05; **P < 0.01; ***P < 0.001; ****P < 0.0001 (WT versus C3aR^−/−^, *WT versus DAF^−/−^, *P* < 0.05). ACR, albumin/creatinine ratio.

In the cBSA model of MN, the formation of anti-cBSA IgG (Figure 1A) is paralleled by a moderate increase in albuminuria (Figure 1B) and BUN (Figure 1C) in BALB/c WT mice, while serum albumin is not significantly different than in vehicle-treated controls at 8 weeks after model induction (Figure 1D).

**Figure 2:**
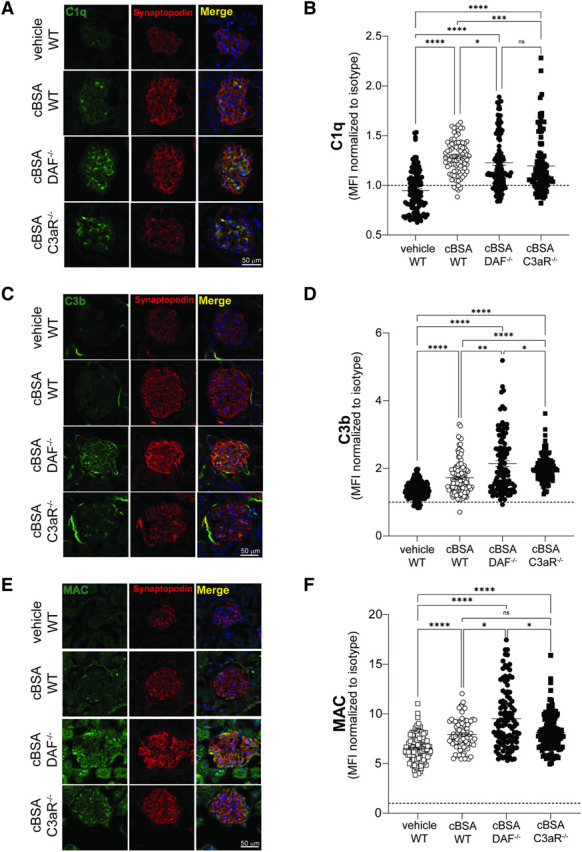
**Glomerular complement deposition differs across cBSA-treated WT, DAF**^**−/−**^**, and C3aR**^**−/−**^ **mice.** Representative images (A, C, and E) and quantification (B, D, and F) of glomerular deposition of C1q (A and B), C3b (C and D), and MAC (C5b-9) (E and F) staining in WT, DAF^−/−^, and C3aR^−/−^ BALB/c mouse at 8 weeks post-cBSA or vehicle control injections. Glomerular fluorescent intensity was quantified relatively to isotype using ImageJ software. Each data point represents a single glomerulus, and all experimental data were verified in three independent experiments (*P < 0.05; **P < 0.01; ***P < 0.001; ****P < 0.0001; ns, not significant). Scale bars 50 *µ*m.

Immunofluorescence analyses of the glomeruli of cBSA-treated mice document deposition of total IgG (Figure 1, E–H). We also noted deposits of complement split products including C1q (Figure 2, A and B), C3b (Figure 2, C and D), and MAC (Figure 2, E and F), suggestive of complement activation through the classical pathway.

Light microscopy shows no or only minimal spike formation of glomerular basement membrane in the Jones methenamine staining, a classic sign of MN immune deposits (Figure 1I). PAS staining did not show relevant changes (not shown).

Altogether, these features indicate that cBSA-induced murine MN recapitulates some of the main characteristics of human disease and is characterized by signs of complement activation.^11^

### Genetic Deletion of DAF Increases Complement Activation and Disease Severity

**Figure 3:**
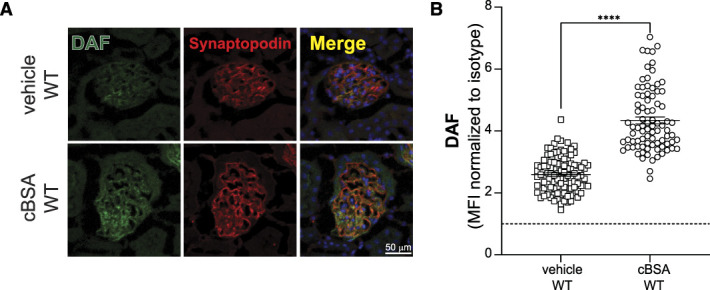
**Glomerular DAF expression in mice.** Representative images (A) and quantification (B) of DAF staining in WT BALB/c mouse glomeruli at 8 weeks post-cBSA or vehicle injections. DAF glomerular fluorescence intensity was quantified relatively to isotype using ImageJ software. At least 30 glomeruli per mouse from two animals were included in the analysis. Each dot represents a single glomerulus, and all experimental data were verified in three independent experiments (****P < 0.0001). Scale bars 50 *µ*m.

We next focused on the role of complement regulator DAF, which is normally expressed on podocyte surface,^17^ in cBSA-induced MN pathogenesis. Immunofluorescence analysis showed that DAF protein was upregulated after the cBSA-MN induction in WT mice (Figure 3), consistent with the finding that in human glomeruli of patients with MN, *DAF* mRNA expression is increased compared with kidneys from healthy donors (Supplemental Figure 1 and Supplemental Methods).

To study the role of DAF in this model, we then tested the susceptibility to cBSA in mice in which DAF was genetically deleted. These mice have no renal abnormalities at baseline.^18^ When we induced cBSA-MN in DAF-deficient animals, we found signs of local complement activation in the glomeruli, as documented by higher C1q (Figure 2, A and B), C3b (Figure 2, C and D), and MAC (Figure 2, E and F) deposition, despite similar levels of circulating anti-cBSA IgG in DAF^−/−^ and WT animals (*P* > 0.999 Figure 1A) and similar IgG glomerular deposition (Figure 1G). Increased complement deposition in DAF^−/−^ mice was associated with higher albuminuria (Figure 1B) and increased severity of the nephrotic syndrome, including increased BUN (Figure 1C) and reduced serum albumin (Figure 1D), than WT animals. Glomerular changes, including spikes of glomerular basement membrane, were also more pronounced in DAF^−/−^ than in WT animals injected with cBSA (Figure 1J).

Overall, these data indicate that DAF is a key regulator of complement activity and a restrainer of disease severity in the cBSA model of MN.

### Genetic C3aR Deletion Reduces Disease Severity in the cBSA-MN Model

Our previous studies^18^ and work by others^22^ show that C3a/C3aR activation in podocytes is a key mediator of podocyte injury and glomerulosclerosis. To test the hypothesis that the same pathway is key in the pathogenesis of the cBSA-induced model of MN, we injected cBSA in C3aR^−/−^ mice. These animals developed similar levels of anti-cBSA IgG antibodies (Figure 1, A and H) and MAC deposition in the glomeruli than WT controls, while deposition of C1q and C3b was higher (Figure 2). In line with our hypothesis, these mice developed significantly lower albuminuria levels than WT animals (Figure 1B). Consistently, histological changes in C3aR^−/−^ mice injected with cBSA were minor (Figure 1K).

Overall, these data suggest that C3a/C3aR signaling plays a pathogenic role in murine cBSA-induced MN.

### Pharmacological C3aR Antagonism Prevents Disease Onset in the cBSA-MN Model

**Figure 4:**
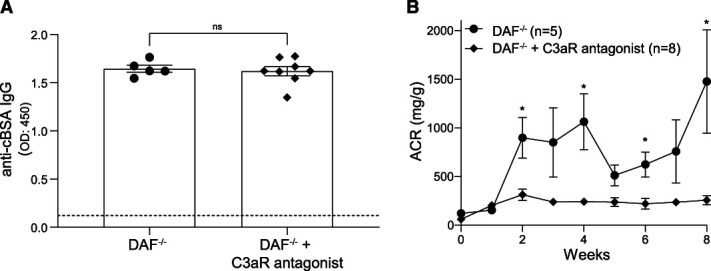
**Pharmacological blockade of C3aR reduces MN disease severity in cBSA-treated DAF**^**−/−**^ **mice.** Serum concentration of anti-cBSA antibodies in DAF^−/−^ BALB/c mice treated with (*n*=8) or without (*n*=5) C3aR antagonist at 8 weeks post-cBSA injections (A). Urinary ACR at weekly intervals in the same mice (B). *P < 0.05; ns, not significant. ACR, albumin/creatinine ratio.

To make our finding more clinically relevant, we treated cBSA-injected DAF^−/−^ mice with a selective C3aR antagonist. Similar to the data with genetic deletion of C3aR, the use of C3aR antagonist did not affect the anti-cBSA IgG concentrations (Figure 4A), but it almost entirely prevented the glomerular disease, as documented by the low albuminuria levels (Figure 4B).

This finding further supports the concept that the main effector mechanism of complement activation in cBSA-induced MN is C3a/C3aR signaling, which is pharmacologically targetable.

## Discussion

Our data suggest that, after immunization with cBSA, mice start forming anti-cBSA IgG that deposit into the glomeruli, where positively charged cBSA binds to the anionic glomerular capillary. The cBSA IgG immune complexes activate complement cascade through the classical pathway which leads to the formation of soluble C3a and membrane-bound MAC. C3a/C3aR signaling in podocytes is the main mechanism of podocyte injury, leading to cytoskeleton rearrangement and foot process effacement.^18^ The glomeruli of cBSA-injected animals show DAF upregulation, which is possibly a compensatory mechanism to restrain uncontrolled complement activation (see the working model in Supplemental Figure 2). Consistently, DAF-deficient mice show increased glomerular complement deposition and disease severity.

Complement system plays a key pathogenic role in MN.^23^ Initial studies with selective depletion or genetic deficiency of components of the distal complement cascade suggested that complement-mediated injury is largely due to the formation of the C5b-9 MAC in the podocyte cell membranes.^13,24^ However, more recent *in vitro* studies suggested an important role for C3a/C3aR and C5a/C5aR1 signaling in podocytes of patients with MN,^12^ and biopsies from individuals with MN show an upregulation of both C3aR and C5aR1 expression in the glomeruli.^12^

In this study, we showed that both genetic deletion of C3aR and its pharmacological inhibition through a selective antagonist prevented disease progression in cBSA-induced MN, indicating a potential therapeutic option for patients with MN. While clinical trials with the anti-C5 eculizumab did not show any significant improvement in patients with MN, currently ongoing clinical trials with APL-2 (pegcetacoplan) (NCT03453619), which specifically binds to C3 and C3b preventing C3a formation, have the potential to show a significant improvement in the outcomes of affected individuals. Recently published data by others suggest that C3a/C3aR signaling in podocytes plays a crucial pathogenic role also in other rodent models of MN.^25^

Intriguingly, despite similar levels of circulating and glomerular anti-cBSA IgG, C3aR^−/−^ mice had higher glomerular deposition of C1q and C3b, possibly because of increased local production of complement components due to the uncoupling of the C3a/C3aR inhibitory signaling.

C5a signaling through C5aR1 has been shown by others to play a role in podocyte injury in MN.^12^ While our data suggest that C3a/C3aR plays a dominant pathogenic role, *ad hoc* studies in cBSA-induced MN model would be needed to clearly dissect the relative contribution of each of these complement receptors.

Mice with cBSA-induced MN display similar histological features to that first described in children with MN by Debiec *et al.*^11^ The origin of cationic BSA remains unclear. Cow's milk is the major source of BSA in young children, and food processing conditions, together with intestinal microbiota metabolism, may lead to pathological modifications of BSA.^26–29^ Regardless from the mechanisms that are responsible for the changes in BSA electrical charge, data in humans with anti–cBSA-MN indicate that complement-activating IgG1 and non–complement-activating IgG4 deposit in the glomeruli and form immune complexes with cBSA.^11^ However, to the best of our knowledge, the pathway of complement activation in humans has not been fully determined. In our study, we found C1q deposition in the glomeruli of cBSA-injected mice, suggesting that complement activation occurs through the classical pathway, initiated by cBSA-IgG immune complexes. However, more studies with mice selectively lacking components of the classical pathway are needed to formally prove this causal link.

Similar to our murine findings, data in humans indicate that cBSA-IgG immune complexes are associated with MAC formation.^11^ The fact that C3aR antagonism reduced disease severity despite MAC deposition in the glomeruli indicates that MAC formation does not represent a major pathogenic role in MN, but additional studies in other models are needed to establish complement effector mechanisms in this condition.

Our results also indicate that the level of complement activation depends on the expression of DAF, but studies are needed to test the role of DAF and other complement regulators in children with cBSA-associated MN and in other adult forms of the disease. These data form the background for testing therapeutic strategies aimed at increasing DAF expression or reducing phosphatase-mediated DAF cleavage in humans with MN. For instance, recent evidence proves that pharmacological inhibition of phospholipase D,^18^ which is responsible for DAF cleavage, reduces glomerular susceptibility to adriamycin injury, supporting this approach.^30^

In sum, our data, generated in a clinically relevant *in vivo* model of MN, indicate that DAF plays a major role in restraining complement activation during MN. Importantly, our data also show that C3a/C3aR activation is the dominant effector mechanism that should be therapeutically targeted to improve the outcomes of affected patients.

## Supplementary Material

SUPPLEMENTARY MATERIAL

## Data Availability

All data are included in the manuscript and/or supporting information.

## Supplemental Material

Supplemental Methods

Supplemental Reference

Supplemental Figure 1. *DAF* gene expression in human glomeruli from participants with MN and healthy controls.

Supplemental Figure 2. Proposed working model for cBSA-induced MN in mice.
