# Supplementary material for: Decay-Accelerating Factor Restrains Complement Activation and Delays Progression of Murine cBSA-Induced Membranous Nephropathy
Source: Kidney360. 2023 Apr 8;4(6):e769–76. doi: 10.34067/KID.0000000000000122 (PMC10371372; doi:10.34067/KID.0000000000000122)
Supplement: SUPPLEMENTARY MATERIAL [file kidney360-4-e769-s001.pdf]

SUPPLEMENTAL MATERIALS

SUPPLEMENTAL METHODS

Nephroseq analysis

Glomerular *DAF* gene expression in humans with membranous nephropathy (MN) was tested using Nephroseq platform (available at: http://www.nephroseq.org/; accessed on June 23, 2021). We analyzed data from one published RNA-sequencing study comparing expression levels of RNA extracted from microdissected glomerular samples from patients with various glomerular diseases, including MN patients (n = 21) and healthy controls (kidney living donors; n = 21) (1).

SUPPLEMENTAL REFERENCE

1. Ju W, Greene CS, Eichinger F, Nair V, Hodgin JB, Bitzer M, Lee YS, Zhu Q, Kehata M, Li M, Jiang S, Rastaldi MP, Cohen CD, Troyanskaya OG, Kretzler M: Defining cell-type specificity at the transcriptional level in human disease. *Genome Res,* 23**:** 1862-1873, 2013 10.1101/gr.155697.113

SUPPLEMENTAL FIGURES

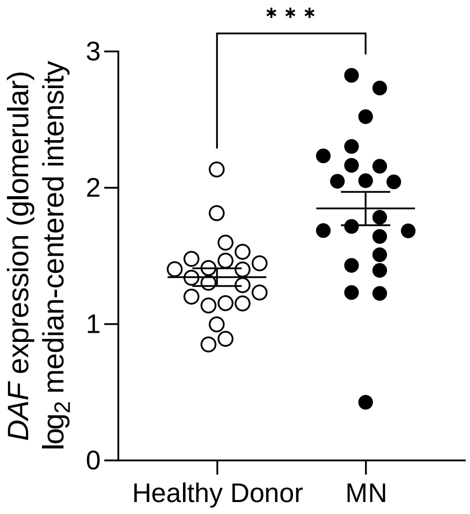

Supplemental Figure 1. *DAF* gene expression in human glomeruli from MN patients and healthy controls.
Glomerular *DAF* gene expression in biopsies obtained from MN patients (n = 21) and healthy controls (kidney living donors; n = 21). Data were extrapolated from the previously published microarray study by Ju et al. (1). ***$P < 0.001$

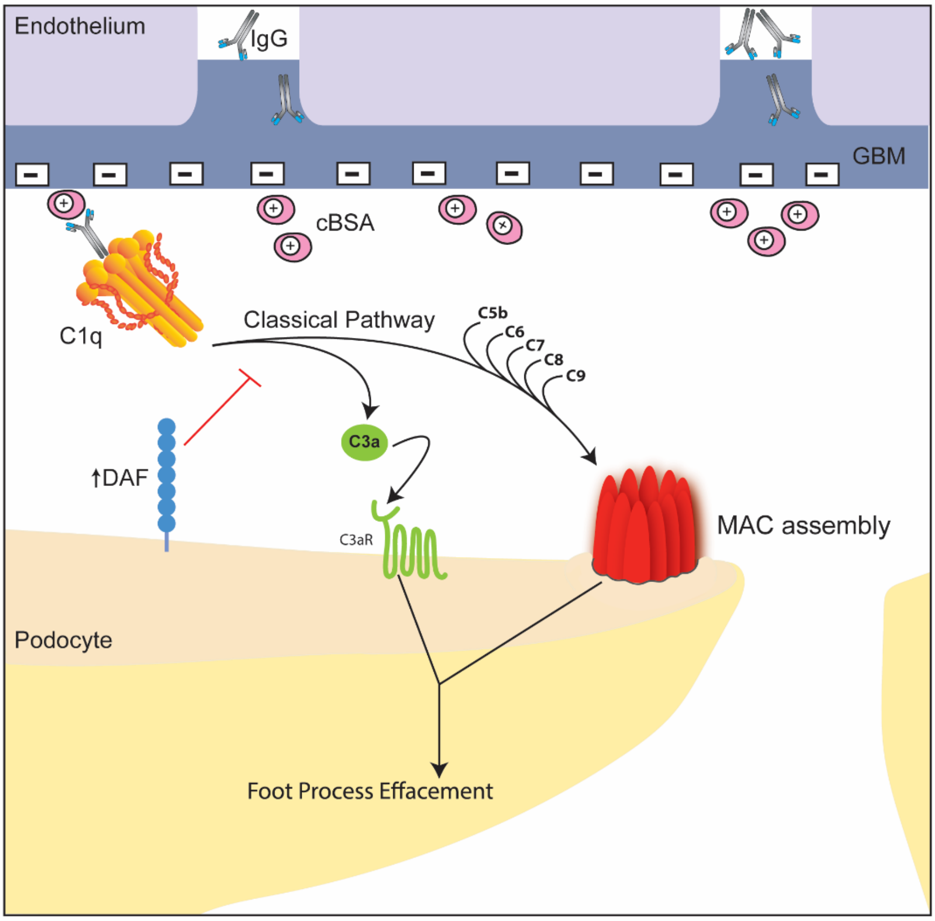

Supplemental Figure 2. Proposed working model for cBSA-induced MN in mice.
Due to its positive electrostatic charge, cationic BSA (cBSA) binds to the anionic glomerular basement membrane (GBM). Anti-cBSA IgG, formed after immunization, deposit in the glomeruli, where they generate cBSA-IgG immune complexes that activate the complement cascade through the classical pathway. Complement regulator DAF is upregulated, possibly as a compensatory mechanism, but this is not sufficient to fully restrain complement activation and C5b-9 membrane attack complex (MAC) formation on podocyte membranes. The main effector mechanism of complement-induced podocyte injury is represented by C3a/C3aR signaling, which leads to cytoskeleton rearrangement and foot process effacement.
